# A novel heterozygous mutation in the hydroxymethylbilane synthase gene in a case with acute intermittent porphyria

**DOI:** 10.5339/qmj.2022.46

**Published:** 2022-10-06

**Authors:** Ritwik Ghosh, Moisés León-Ruiz, Sona Singh Sardar, Dinobandhu Naga, Dipayan Roy, Tapas Ghosh, Souvik Dubey, Julián Benito-León

**Affiliations:** ^1^Department of General Medicine, Burdwan Medical College & Hospital, Burdwan, West Bengal, India E-mail: jbenitol67@gmail.com; ^2^Section of Clinical Neurophysiology, Department of Neurology, University Hospital “La Paz,” Madrid, Spain; ^3^Department of Biochemistry, All India Institute of Medical Sciences (AIIMS), Jodhpur, Rajasthan, India.; ^4^Indian Institute of Technology (IIT), Madras, Tamil Nadu, India; ^5^School of Humanities, Indira Gandhi National Open University, New Delhi, India; ^6^Department of Anatomy, Burdwan Medical College & Hospital, Burdwan, West Bengal, India; ^7^Department of Neuromedicine, Bangur Institute of Neurosciences (BIN), Kolkata, West Bengal, India; ^8^Department of Neurology, University Hospital “12 de Octubre”, Madrid, Spain; ^9^Centro de Investigación Biomédica en Red Sobre Enfermedades Neurodegenerativas (CIBERNED), Madrid, Spain; ^10^Department of Medicine, Complutense University, Madrid, Spain

**Keywords:** Acute Intermittent Porphyria, Hydroxymethylbilane Synthase Mutation, p.Arg173Trp

## Abstract

Porphyrias are rare metabolic disorders caused by inherited or acquired enzymatic defects in the heme biosynthetic pathway. They are grouped into acute hepatic porphyrias and photocutaneous porphyrias. Acute intermittent porphyria, the most prevalent subtype of acute hepatic porphyrias, is caused by a mutation in the hydroxymethylbilane synthase gene. In this work, a case of a 13 year-old Indian female presenting with multi-organ involvement (Neurological: episodic seizures, behavioral abnormalities, acute onset progressive flaccid-motor quadriparesis, multiple cranial nerve palsies, respiratory paralysis, dysautonomia, and posterior reversible encephalopathy syndrome; Gastrointestinal: recurrent attacks of abdominal pain, nausea/vomiting, isolated transaminitis, and acute pancreatitis; and Renal: metabolic alkalosis and refractory dyselectrolytemia) which resulted in significant diagnostic dilemmas. She was eventually diagnosed as a case of acute intermittent porphyria harboring a novel hydroxymethylbilane synthase gene mutation (p.Arg173Trp).

## Introduction

Porphyrias are rare metabolic disorders caused by inherited or acquired enzymatic defects in the heme biosynthetic pathway^1^. They are grouped into acute hepatic porphyrias and photocutaneous porphyrias^1^. Amid them, hepatic porphyrias stem from hepatic overproduction of the porphyrin precursors, affecting the nervous system due to the accumulation of byproducts of a metabolic pathway that enter the circulation and are excreted into urine and bile^1,2^.

Acute intermittent porphyria (AIP) is the most prevalent subtype of acute hepatic porphyrias, accounting for around 80% of all symptomatic cases^1^. In AIP, induction of the hepatic delta-aminolevulinic acid (δ-ALA) synthase-1 results in the accumulation of neurotoxic heme-intermediates δ-ALA and porphobilinogen, affecting the nervous system and other organs, and is potentially life-threatening^1,2^.

In a European 3-year prospective study, the prevalence of clinically overt AIP was 5.4 cases per million^3^. Nevertheless, large-scale epidemiological studies on the prevalence of AIP are rare in India and are mainly based on small case series mostly from Northwestern^4^. In a cross-sectional study of the rural community (n = 2,385) of Kumhars of Bikaner district of Western Rajasthan, the prevalence of AIP was 1.16%, being more frequent in females (2:1), with the majority of cases (38.9%) in the age group of 20-29 years, and the average age of manifestation was 24.5+/- 4.8 years^5^.

Mutation in the hydroxymethylbilane synthase (HMBS), also known as porphobilinogen deaminase, a gene that causes AIP, is a prevalent mutation in Western populations (1 carrier per 2000 persons). However, acute flares appear in < 10% of the at-risk population, suggesting a significant role of environmental factors and possibly genetic modifiers^1^. Most AIP cases carry the founder mutation c.669_698del30 in the HMBS gene. Besides, higher penetrance has been linked to specific mutations, such as R173W and W198X^6^, and particular mutations are more frequent among patients with multiple or recurrent attacks (e.g., those encoding p.R149X, p.R173Q)^7^.

We report a case of a 13 year-old Indian girl who presented with multi-organ involvement (Neurological: episodic seizures, behavioral abnormalities, acute onset progressive flaccid-motor quadriparesis, multiple cranial nerve palsies, respiratory paralysis, dysautonomia, and posterior reversible encephalopathy syndrome [PRES]; Gastrointestinal: recurrent attacks of abdominal pain, nausea/vomiting, isolated transaminitis, and acute pancreatitis; and Renal: metabolic alkalosis and refractory dyselectrolytemias) which resulted in significant diagnostic dilemmas. She was eventually diagnosed as a case of AIP harboring a novel hydroxymethylbilane synthase (HMBS) gene mutation (p.Arg173Trp).

Her father provided informed consent for the publication and data usage concerning the case report.

## Case Report

A 13 year-old endotracheally intubated (connected with an artificial manual breathing unit) girl from rural West Bengal (India) was admitted to the intensive care unit because of neuromuscular respiratory paralysis. She was rapidly put on mechanical ventilation without using muscle relaxants. After hemodynamic stabilization, her caregivers were called for detailed history taking. According to them, she was born out of non-consanguineous wedlock and remained healthy until last year, when she developed the symptoms listed in Table 1.

We got some exciting leads about the disease trend by scrutinizing previous historical records and conducting laboratory tests in different timelines. Historical trends divulged a definite history of intake of estrogen-progesterone hormone-containing pills during each attack on premenstrual days, followed by worsening abdominal pain, aggravation of behavioral abnormalities, and sometimes “seizure-like episodes.” These episodes were often (and not always) associated with reddish urine discoloration. Last but not least, blood pressure fluctuations were observed during each attack. Laboratory trend has been summarized in Table 1.

The family members (her father, paternal uncle/aunt, and cousin; no one from her maternal side who can be considered a first-degree relative was alive) revealed that the patient's mother also had a similar illness with four similar episodes of abdominal pain followed by abrupt-onset partly reversible flaccid quadriparesis. She died without a confirmed diagnosis in the last attack following the patient's birth.

Complementary tests initially performed are listed in Table 1. Detailed neurological examination at and after admission during the last hospitalization is included in Table 1. A few differential diagnoses were considered (Table 2): AIP, complicated Gitelman syndrome (and similar genetic channelopathies), recurrent Landry-Guillain-Barré-Strohl syndrome, and hereditary motor-sensory neuropathy variant with episodic worsening.

**Figure 1.**
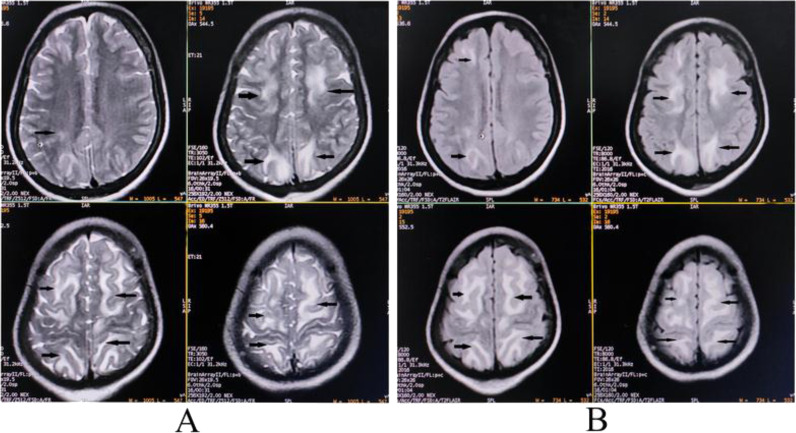
MRI of the brain revealing hyperintensity on tbl2-weighted (A) and tbl2-FLAIR, (B) images involving bilateral frontal and parieto-occipital grey-white interface (arrows), suggestive of PRES.

She was treated symptomatically with careful monitoring of fluid and acid-base balance. Her acid-base disturbances and dyselectrolytemia were gradually corrected with water restriction and intravenous magnesium and calcium supplementation. Keeping a possibility of AIP in the backdrop, she was given a high-dose intravenous dextrose infusion. She was also prescribed intravenous magnesium sulfate, calcium gluconate, sedative (intravenous dexmedetomidine), brivaracetam (150 mg/day first intravenously; then orally through a nasogastric tube), propranolol (orally for counteracting cardiac dysautonomia), tolvaptan (15 mg/day orally for an inappropriate secretion of antidiuretic hormone), and adequate nutritional supplementations. She was put on a weaning trial from day four onwards. After seven days, she could eventually be weaned off ventilator support and immediately went for magnetic resonance imaging of the brain and spinal cord, revealing lesions suggestive of PRES (Figure 1). After weaning from the ventilator, she could generally communicate with us, though in a much-changed voice (due to weakness of bulbar muscles and bifacial paralysis) and had wasting of tongue and facial muscles and deglutition difficulties. The Ryle's nasogastric tube for feeding (diet was modified, keeping the possibility of AIP in mind) was maintained, and bedside physiotherapy was continued.

Unfortunately, she died from ventilator-associated pneumonia and subsequent sepsis. A blood sample was sent for clinical exome sequencing after getting proper standard informed consent for such testing from the legal guardian and the institutional ethics committee. The DNA extracted from blood was used to perform targeted gene capture. The libraries were sequenced to mean >80-100X coverage on the Illumina sequencing platform. The sequences obtained were aligned to the human reference genome (GRCh38.p13) and analyzed for removing duplicates, recalibration, and realignment of indels using the Sentieon (v201808.07) aligner^8^. Gene annotation of the variants was performed against the Ensembl release 99 human gene model^9^. Only non-synonymous (calculated using algorithms such as PolyPhen-2, SIFT, LRT, and MutationTaster2) and splice site variants detected in the clinical exome panel consisting of 6120 genes were used for clinical interpretation.

**Figure 2.**
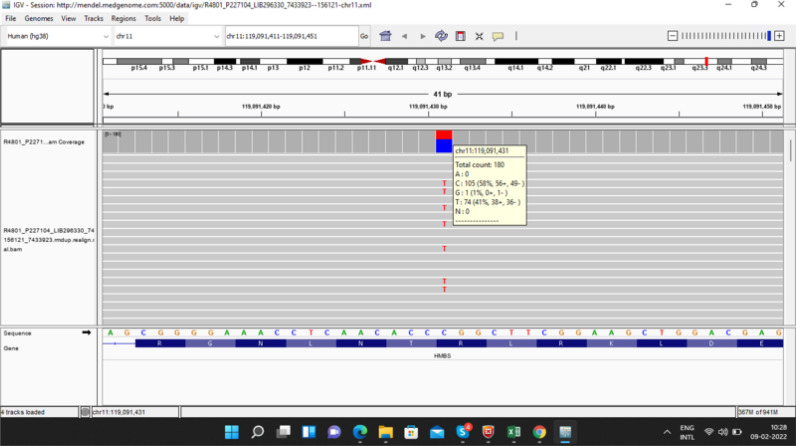
Integrative genomic viewer depicting visualization of the pathogenic variant c.517C>T (p.Arg173Trp) in exon 9 of the HMBS gene (chr11:g.119091431C>T; depth: 175x).

Posthumously, the genetic report disclosed a new heterozygous, autosomal dominant mutation (missense variation in exon 9) in the HMBS gene, which produces the amino acid substitution of Tryptophan for Arginine at codon 173 (p.Arg173Trp) (Figure 2 and Table 1).

## Discussion

The p.Arg173Trp variant has not previously been reported in AIP patients; it lies in the HMBS protein's CBL proto-oncogene N-terminal domain 1^8^. Experimental studies have shown that this missense change abrogates HMBS activity and causes conformational instability of the protein^10^. The ClinVar database considers this variant pathogenic^11^. Moreover, different *in silico* predictors (PolyPhen-2, SIFT, and LRT) have classified this variant as pathogenic.

Flares in AIP are more often seen in females. They consist of severe diffuse abdominal pain (the cardinal symptom of AIP), muscle weakness, autonomic neuropathy (e.g., arterial hypertension, tachycardia, nausea, vomiting, constipation, and sometimes PRES), and changes in mental status. Complications usually result from severe episodes of acute attacks, including paralysis, hyponatremia (with risk of central pontine myelinolysis), seizure-like spells, and coma. An acute attack may also trigger neuropsychiatric symptoms such as confusion, hallucinations, anxiety, and psychosis. Most symptomatic patients have only a few episodes during their lifetime and are generally self-limiting, but 8% have recurrent seizures. They warrant urgent medical attention and sometimes prolonged hospitalization and rehabilitation^1^.

Recurrent self-limiting quadriparesis, clinically mimicking Landry-Guillain-Barré-Strohl syndrome, is an essential diagnostic clue of AIP if associated with multiple cranial nerve involvement, respiratory paralysis, and other symptoms such as acute-onset abdominal pain, seizures, new-onset behavioral abnormalities/personality changes, and persistent autonomic dysfunction. AIP is still an underrecognized entity in India, probably due to the low index of clinical suspicion and difficulties in confirming the diagnosis. The exact diagnosis of this entity is much appreciated to avoid inadvertent drug exposures (like oral hormone-containing pills in this case) that can further precipitate acute attacks. Also, family and medication history should be searched meticulously. AIP should be included within the differential diagnoses for unexplained abdominal pain and neuropsychiatric symptoms to raise clinicians’ awareness and prevent misdiagnosis^1^. Acute relapsing pancreatitis, recurrent transaminitis, PRES, and inappropriate secretion of antidiuretic hormone, in isolation or combination, have been rarely reported previously in several cases of AIP^12-14^. However, these clinical-radiological associations in a single point, perhaps, have not been previously reported, making this unique. These rare, but established, associations make the diagnosis difficult and compel the clinicians to consider several other close differential diagnoses like this case. Metabolic alkalosis, recalcitrant hypokalemia, hypomagnesemia, hyponatremia with recurrent quadriparesis, and positive family history can also be found in untreated and complicated Gitelman syndrome, exceptionally rarely associated with chronic pancreatitis^15^. Genetic testing remains conclusive to confirm or refute the diagnosis in such situations.

The treatment of AIP remains challenging even today. Acute pain management has scarcely been studied, with a striking lack of information through evidence-based recommendations^16^. Current acute treatment options include removing triggering factors and treatment with intravenous opioids, glucose, and hemin^16^. Preventive therapies include hormone suppression, off-label prophylactic hemin, and liver transplantation. On November 20, 2019, the latest therapeutic approach was the FDA-approved givosiran for adults with AIP. It is a 2.5 mg/kg once-monthly subcutaneously administered RNA interference targeting hepatic δ-ALA synthase one messenger RNA (mRNA), preventing the accumulation of δ-ALA and porphobilinogen. It is particularly suitable for patients with severe recurrent attacks^17^. While generally well tolerated with an appropriate safety profile, it may increase the risk of some adverse events (raised serum aminotransferase levels, changes in serum creatinine levels, and the estimated glomerular filtration rate and injection-site reactions.)^17-19^. This is probably the first reported case of AIP caused by the hydroxymethylbilane synthase p.Arg173Trp variant. Hence, this scarce variant should be included in the genetic diagnostic protocol of AIP for prompt diagnosis and proper treatment of this potentially lethal disease.

## Ethics Statement

We thank the patient's caregiver for granting permission to publish this case.

## Figures and Tables

**Table 1:** Summary of clinical history, differential diagnoses, and ancillary investigations.

	Clinical history		Investigations

Since menarche	• She often complained of central abdominal pain, nausea, and vomiting, which became severe, especially during her perimenstrual days. She was diagnosed with primary dysmenorrhea and treated with hormonal pills (estrogen and progesterone), proton-pump inhibitors, and non-steroidal anti-inflammatory drugs. • Surprisingly, according to her caregivers, her pain increased almost every time she took the medications above. She often had to get admitted to the hospital to manage the pain.	During every hospital/nursing home admission	• Marked euvolemic hyponatremia (Na^+^ levels between 109 to 119 mEq/L). • Mild to moderate hypokalemia (K+ levels between 1.9 to 3.1 mEq/L). • Mild hypocalcemia. • Isolated hypertransaminasemia (transaminase levels between 3-10 times upper limits of normal ranges; without other liver function test changes). • Raised pancreatic enzymes (amylase and lipase levels between 2-5 times the upper limits of normal ranges. • Definite anatomical (ultrasound scan and computed tomography) features of pancreatitis. • Sub-clinical hyperthyroidism (low to lower normal TSH with normal unbound tbl4 and tbl3 levels). • Loss of sinus arrhythmia on serial electrocardiography.

Last six months	• On three occasions, she had "seizure-like episodes," changes in behavior and personality, and severe abdominal pain after ingestion of hormonal pills. She was referred to two neurologists and psychiatrists who put her on antipsychotics. • As the "seizure-like episodes" continued in the next few months, one neurologist added valproic acid and increased the dose of antipsychotics considering it to be some "psychiatric illness."	During the last hospitalization	• Arterial blood gas analysis and relevant laboratory reports were remarkable for metabolic alkalosis and dyselectrolytemia (hyponatremia, hypokalemia, hypocalcemia, and hypomagnesemia). • Blood urea nitrogen serum and creatinine levels were normal. • The complete blood cell count was unremarkable. • Thyroid function tests revealed low TSH with low-normal unbound tbl4, decreased unbound tbl3, and high reverse tbl3 levels, suggesting non-thyroidal illness syndrome. • Blood glucose levels were normal and were being continuously monitored.

Four months before the hospital admission	• She was admitted to a private hospital for an episode of severe abdominal pain, vomiting (following intake of the same		• Consecutive urinalysis failed to reveal hematuria/hemoglobinuria, but qualitative and quantitative tests for

	medications), "seizure-like episodes," personality changes, bizarre behavior, and abrupt-onset flaccid weakness of all four extremities. • Within one week, she recovered significantly and was discharged as a diagnosed case of dyselectrolytemia with hypokalemic paralysis. • However, she needed almost one and a half months, even after discharge from the hospital, for "complete recovery." • The treating consultants told the caregivers that the girl had an episode of pancreatitis due to psychiatric medications (probably caused by valproic acid), resulting in abdominal pain, nausea, vomiting, hyponatremia, and hypokalemia, which led to the seizures and rapidly-reversible quadriparesis.		porphobilinogen in urine (column chromatography) gave positive results. • Clinical euvolemic status, serum and urine osmolality, and serum and urine sodium levels pointed towards a syndrome of inappropriate antidiuretic hormone secretion. • Liver function tests revealed an isolated rise in transaminases by tenfold the normal's upper limits. • Serum lipase and amylase were also raised. • Of note, nerve conduction studies revealed acute motor-predominant polyneuropathy with features of a primary axonopathy with a secondary demyelinating pattern. • The cerebrospinal fluid study revealed significant albuminocytologic dissociation (protein 3.5 g/L, glucose 70 mg/dL, and four cells per microliter). Serologies for hepatitis (A, B, C, and E), HIV (1 and 2), Lyme borreliosis, tuberculosis, syphilis, scrub typhus, and SARS-CoV-2 were negative.

During the last hospitalization	At admission: • She was conscious and could communicate with us with side-to-side head nodding and through the movement of eyelids. • Autonomic function tests revealed significant dysautonomia. • She had severe (mMRC strength 1/5) lower motor neuron type quadriparesis with bilateral lower cranial (IX, X, XI, XII) facial nerve palsies. • She also had unexplained tachycardia and spiking blood pressure due to autonomic instability.		• Anti-neuronal antibodies (non-paraneoplastic and paraneoplastic) and anti-ganglioside antibodies were negative; tests for connective tissue disorders and autoimmune vasculitis, including Bechet's disease and sarcoidosis were negative as well. • Bedside monitoring revealed loss of heart rate variability on electrocardiography. • An electroencephalogram was normal. • Clinical exome sequencing: disclosed a heterozygous, autosomal dominant missense variant in exon 9 of the HMBS gene (chr11:g.119091431C>T;

	• The urine bag showed an adequate amount of urine, which turned reddish after some time. After admission: • Her neurological deficit remained the same, except good improvement of the respiratory muscle weakness and mild improvement in quadriparesis (MRC grade 2+/5 in all four limbs), and she had no breakthrough seizures. • After another week, she again had an episode of acute abdominal pain, altered sensorium, respiratory depression, and increased frequency of focal "seizure-like episodes." • She again required ventilator support. • Since there was no improvement in the respiratory parameters, a tracheostomy was done, and a ventilator was connected to the intra-tracheal tube.		Depth: 175x) that results in the amino acid substitution of Tryptophan for Arginine at codon 173 (p.Arg173Trp; ENST00000652429.1).


**Table 2:** Close differential diagnoses with points in favor and odds.

Differential diagnoses	Points in favor and odds

Gitelman syndrome or similar channelopathies	*In favor:* Recurrent hypokalemia during each attack, mild hypocalcemia, metabolic alkalosis with recurrent episodes of acute flaccid quadriparesis with one episode of arguable rapid neurological response to dyselectrolytemia correction, family history of similar neurological disease *Odds*: Associated abdominal pain, seizures, psychiatric issues, pancreatitis, transaminitis, dysautonomic features, posterior reversible encephalopathy syndrome, non-thyroidal illness syndrome, cranial nerve involvements, associated significant hyponatremia (and syndrome of inappropriate antidiuretic hormone secretion), albuminocytological dissociation in the cerebrospinal fluid study, extremely poor and slower recovery of current neurological deficits, positive urine porphobilinogen tests, genetic analysis suggestive of acute intermittent porphyria and not of any channelopathy

Recurrent Landry-Guillain-Barré-Strohl syndrome	*In favor:* Episodic acute flaccid quadriparesis with involvement of lower cranial nerves, bilateral facial nerves, dysautonomic features, and posterior reversible encephalopathy syndrome, albuminocytological dissociation in cerebrospinal fluid study, positive family history, previous episode of auto-recovery *Odds:* Associated abdominal pain, seizures, psychiatric issues, pancreatitis, transaminitis, non-thyroidal illness syndrome, associated significant hyponatremia (and syndrome of inappropriate antidiuretic hormone secretion), extremely poor and slower recovery of current neurological deficits, positive urine porphobilinogen tests, genetic analysis suggestive of acute intermittent porphyria and not of any genetic variants of Landry-Guillain-Barré-Strohl syndrome

Hereditary motor-sensory neuropathy variant with episodic worsening	*In favor:* Episodic acute flaccid quadriparesis with positive family history, previous episode of quick auto-recovery *Odds:* Associated abdominal pain, seizures, psychiatric issues, pancreatitis, transaminitis, dysautonomic features, posterior reversible encephalopathy syndrome, non-thyroidal illness syndrome, cranial nerve involvements, associated significant hyponatremia (and syndrome of inappropriate antidiuretic hormone secretion), albuminocytological dissociation in the cerebrospinal fluid study, extremely poor and slower recovery of current neurological deficits, positive urine porphobilinogen tests, genetic analysis suggestive of acute intermittent porphyria and not of any genetic HMSN variants

